# Analytical and clinical concordance of free light chain assay

**DOI:** 10.1016/j.plabm.2018.e00112

**Published:** 2018-12-05

**Authors:** Andrew Smith, Alan H.B. Wu

**Affiliations:** Clinical Chemistry Laboratory, San Francisco General Hospital, 1001 Potrero Ave., San Francisco, CA 94110, United States

**Keywords:** Free light chain, Multiple myeloma, Immunoglobulins

The analysis of serum for the presence of free light chains has become an important adjunct to testing by serum protein electrophoresis (SPE) and immunofixation electrophoresis (IFE) [1]. The first commercial free light chain assays using polyclonal reagents was released in 2001 by Binding Site Inc. (Freelite™). Subsequently, in 2011, Siemens released monoclonal-based free light chain assays. The interest in free light chain testing has increased with a more than doubling of the subscription rates to the College of American Proficiency Testing Survey from 2010. We validated the Diazyme free light chain assay, a latex particle enhanced immunoturbidimetric assay that uses polyclonal antibodies and was adapted to the Siemens Advia 1800 analyzer. As this assay was not FDA approved on this analyzer, we considered it as a “lab developed test.” We conducted a clinical concordance study compared to the Freelite assay as the predicate.

A total of 222 samples were obtained from 175 different patients submitted for routine SPE and IFE (agarose gel-based from Sebia), and free light chain analysis as part of a routine workup for multiple myeloma. There were 131 patients with no myeloma (including those with monoclonal gammopathy of unknown significance, MGUS) and 42 patients exhibiting a monoclonal band, including 4 with light chains disease, and 2 with amyloidosis. The majority of the myeloma patients had IgG-kappa (25 patients) with at least one of each of the other 5 major subtypes (no IgD or IgE myelomas). This study was conducted as part of a quality assurance and validation study for potential implementation of this assay in our hospital laboratory. Samples were permanently de-identified once the required medical information was extracted. As this was not a research study, institutional review board approval was deemed to be unnecessary.

The recommended reference range for the Diazyme assay is 2.37–20.73 mg/L for free kappa (κ) and 4.23–27.69 mg/L for free lambda (λ), and 3.30–19.40 and 5.71–26.30 mg/L for the Binding Site free κ and free λ assays, respectively. The reference range for κ /λ for the Diazyme assays is 0.22–1.74, and 0.26–1.65 for the Binding Site Assay.

The precision of the Diazyme assay for 27 days for two levels of controls (nominal values 1.50 and 3.21 mg/L for κ and 2.49 and 4.93 mg/L for λ) was 6.4% and 3.2% for κ free light chains (level 1 and 2, respectively) and 3.2% and 1.6% for free λ light chains, respectively. The correlation between the two assays for free κ and λ was produced a linear correlation equation of y = 1.03 × −18.9 mg/L, r = 0.95 and y = 1.06 × +4.1 mg/L r = 0.97, respectively (the log–log scale representation is shown in Fig. 1A and B, Medcalc Inc.). Five points were removed from the λ correlation because they were below the Diazyme assay's limit of quantitation of 4.23 mg/L. The regression for the kappa to lambda ratio was y = 0.32 × +1.90, r = 0.97 (Fig. 1C). The few significant outliers that were observed (e.g., points A–E, Fig. 1A–C) were all outside the reference range for these analytes (none were removed from the regression equations). A review of the cases showed that none of these would have resulted in a change the diagnosis of myeloma, which at our institution, is largely made on the basis of the clinical presentation, the immunofixation electrophoresis finding, conducted on all myeloma and MGUS patients, and bone marrow biopsies where appropriate. Serum free light chains are usually used to monitor disease progress and remission.

**Fig. 1:**
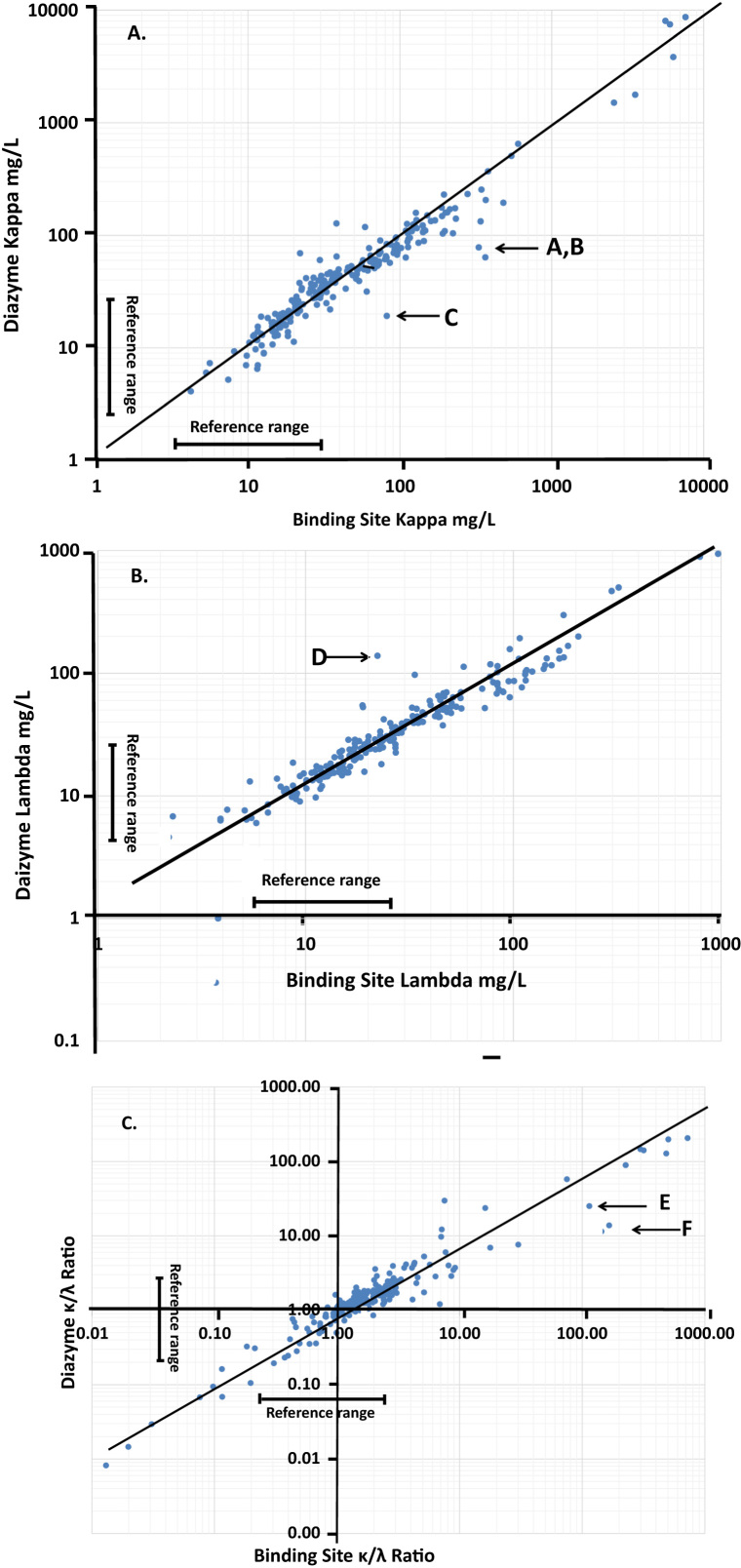
Correlation of the Diazyme vs. Binding Site A) free kappa, B) free lambda, and C) K/λ.

Based on manufacturer's claims, the Diazyme assay had a wider analytical measurement range for free κ and free λ (150 and 200 mg/L, respectively than the Binding Site assay (56.2 and 74.8 mg/L respectively) resulting in fewer numbers of samples that required dilution after the initial test (9.5% and 1.4% for Diazyme, respectively versus 34% and 18% for Binding Site, respectively).

Using the recommended reference intervals for the two kits, the degree of concordance was 99% for free κ, with both discordant results being near the cutoff concentration (i.e., within 10% of the cutoff limit to account for assay imprecision). The concordance for the free λ assay was 90%, with most discordant results being near the cutoff concentration. If the Diazyme cutoff upper reference range is raised to 32.0 mg/L, the degree of concordance increase to 94% (all were positive by the Diazyme and negative by Binding Site assays). Among the discordant results, all but 2 patients (3 samples total) had result values near the cutoff concentration for the two assays. A review of the medical records showed that myeloma was present in both patients.

For the κ/ λ ratio, the degree of concordance using the manufacturer's recommended reference interval was 79%, with 47 discrepancies. For the purpose of this study, we varied the Diazyme reference range by lowering it from 0.22–1.74 to 0.19 to 1.50. The degree of concordance improved to 86% (1 low discordance and 29 high discordance). Among the discordant results, all but 6 had results near the cutoff ratio for these two assays (i.e., within the imprecision of the assay, of roughly 10%). All were positive for the Binding Site assay ratio and negative for the Diazyme assay ratio (3 with and 3 without myeloma). These discrepant patients were not the same as the two discordant patients using the free λ test. Use of a lower Diazyme cutoff would have resulted in more false positive results. We are not suggesting an alteration in the reference range recommended by Diazyme and approved by the FDA, as a reference range study was not conducted. By altering the cutoff, we wanted to show that the discordance between the two assays was likely imprecision of the assay at cutoff limits.

Previous reports have demonstrated a higher degree of clinical discordances between different manufacturers of free light reagents. In three of these studies, monoclonal antibodies were used as the reagent [2], [3], [4], one non-commercial assay and two Siemens assay respectively. There were some discrepancies in the results of these two assays with some discussion written by scientists at Binding Site [5], [6]. In one of the rebuttals suggested that the cause of discrepancies could be the presence of amyloidosis or free light chains disease [5]. The discordant patients reported in this study were not among the 2 patients with amyloidosis or the 4 patients suffering from free light chain disease. Overall, we demonstrated less discordance of results probably because of the use of polyclonal antibodies in both assays. Given the heterogeneity of multiple myeloma expression, i.e., diversity within the variable domain region, use of polyclonal antibodies should capture more variant immunoglobulin forms. A future study is warranted to determine if differences between monoclonal vs. polyclonal-based free light chain assays have clinical relevance.

It is often necessary for clinical laboratories to change the reagents used for a laboratory test or tests if different instruments are used, there is an alteration in the availability of reagents such as with an FDA recall, or for economic reasons. The guidelines for use of free light chains for myeloma diagnosis and monitoring were based on results obtained from the predicate assay (Binding Site). If there is concordance of results with a new manufacturer of reagents, it may be possible to substitute results of the new assay against the previous one following some clinical validation, as was performed in this study. However, each laboratory must conduct some validation studies for use of the new assay prior to use within their practice. This may be accompanied by a change in the reference ranges. The use of values approved by the FDA is preferred unless the laboratory has the means and rationale to conducting their own reference ranges studies. Simply adopting recommended new reference ranges are less of an issue for diagnostic tests where results are used in cross section, than for tests that are longitudinally used for monitoring disease. Changing manufacturers of tumor markers can be difficult due to heterogeneity of the marker, e.g., different degrees of glycosylations, results in a high degree of discordance [7]. For serial monitoring of myeloma patients, clinical laboratories switching from one assay to another may consider saving samples tested on prior free light chain reagents in order to “re-baseline” results from the new reagents. As discordant results are almost always present when changing reagents or methods, this approach would reduce misinterpretation of test results should outliers be present.

## Funding

This work was funded by Diazyme Inc., United States.
